# Smoking: NRT in Pregnancy

**Published:** 2007-05

**Authors:** Carol Potera

Despite health warnings, at least 11% of U.S. women smoke during pregnancy. Smoking doubles the risk for low-birth-weight babies and raises the risk for preterm delivery and sudden infant death syndrome; premature and low-birthweight infants face increased odds for behavioral and learning problems and chronic disabilities such as cerebral palsy. Physicians typically have not viewed nicotine replacement therapy (NRT) as a safe alternative for pregnant women who can not or will not quit smoking. But a new study challenges that stance.

One reason physicians are reluctant to treat pregnant smokers with NRT stems from the findings of 20-year-old studies in which pregnant rats received nicotine doses well above those experienced by heavy smokers. Indeed, pregnant women would have to smoke up to 500 cigarettes a day to mirror those doses, according to Shabih Hasan, a neonatologist at the University of Calgary’s Health Sciences Centre. In contrast, Hasan and colleagues treated pregnant rats with nicotine doses that replicate levels measured in the blood of pregnant women who smoke. The goal of Hasan’s study was to study the effects of nicotine on litter size and pup weight, given that fetal growth restriction is a known effect of maternal smoking.

At a nicotine dose of 2 mg/kg/day, both litter size and pup birth weights were normal. Even in the second and third trimesters, when clearance of nicotine slows, the rat mothers and fetuses stayed healthy. “High blood nicotine levels have no apparent effect on pregnancy in rats, including fetal weight gain,” says Hasan, who reported the results in the 1 January 2007 issue of *Toxicology and Applied Pharmacology*.

Cigarette smoke contains 4,700 chemicals, including numerous toxicants and carcinogens. Hasan says short-term use of NRT during pregnancy could decrease exposure of mothers and fetuses to potentially more harmful components of cigarette smoke.

A Danish trial published in the December 2000 issue of *Obstetrics & Gynecology* provided some early clues to the safety of NRT on pregnancy. Half of 250 pregnant smokers received NRT as skin patches, and their babies’ birth weights averaged 186 g higher than those born to women using placebo patches. Larger clinical studies are needed to monitor the safety and investigate the effectiveness of NRT in pregnant smokers, including long-term follow-up of mothers and babies, says Hasan.

“Logically, nicotine replacement should be safer than smoking, but there is no direct evidence that this is so,” says Tim Coleman, an associate professor of primary care at the University of Nottingham. In March 2007, Coleman and his colleagues started recruiting more than 1,000 pregnant smokers in the United Kingdom for a double-blind, placebo-randomized trial of NRT in pregnancy. The trial, funded by the British National Health Service and the largest of its type ever conducted, will investigate the specific impact of nicotine on the developing human fetus and subsequently in infancy. Volunteers will receive nicotine or placebo skin patches and behavioral support to quit smoking. The women and babies will be followed for two years to evaluate medical and behavioral outcomes, according to the protocol described in the January 2007 *BMC Health Services Research*.

Meanwhile, pregnant smokers should not treat themselves with over-the-counter NRT products. “Unless blood, urine, and/or saliva levels of nicotine or its metabolites like cotinine are measured and closely monitored, women could inadvertently overdose themselves by continuing to smoke while using nicotine replacement products,” Hasan cautions. Quitting smoking remains by far the best option.

